# Chemical Compositions, Mosquito Larvicidal and Antimicrobial Activities of Essential Oils from Five Species of *Cinnamomum* Growing Wild in North Central Vietnam

**DOI:** 10.3390/molecules25061303

**Published:** 2020-03-12

**Authors:** Do N. Dai, Nguyen T. Chung, Le T. Huong, Nguyen H. Hung, Dao T.M. Chau, Nguyen T. Yen, William N. Setzer

**Affiliations:** 1 Graduate University of Science and Technology, Vietnam Academy of Science and Technology, 18-Hoang Quoc Viet, Cau Giay, Hanoi 10072, Vietnam; chungpuhoat@gmail.com; 2 Faculty of Agriculture, Forestry and Fishery, Nghe An College of Economics, 51-Ly Tu Trong, Vinh City 4300, Nghe An Province, Vietnam; 3 School of Natural Science Education, Vinh University, 182 Le Duan, Vinh City 4300, Nghệ An Province, Vietnam; lehuong223@gmail.com (L.T.H.); nguyenthiyenth92@gmail.com (N.T.Y.); 4 Center for Advanced Chemistry, Institute of Research and Development, Duy Tan University, 03 Quang Trung, Da Nang 5000, Vietnam; nguyenhuyhung@duytan.edu.vn; 5 Institute of Environmental Biochemistry, Vinh University, 182 Le Duan, Vinh City 4300, Nghệ An Province, Vietnam; daochau27@gmail.com; 6 Aromatic Plant Research Center, 230 N 1200 E, Suite 100, Lehi, UT 84043, USA; 7 Department of Chemistry, University of Alabama in Huntsville, Huntsville, AL 35899, USA

**Keywords:** Lauraceae, *Aedes aegypti*, *Aedes albopictus*, *Culex quinquefasciatus*, antibacterial, antifungal

## Abstract

Members of the genus *Cinnamomum* (Lauraceae) have aromatic volatiles in their leaves and bark and some species are commercially important herbs and spices. In this work, the essential oils from five species of *Cinnamomum* (*C. damhaensis*, *C. longipetiolatum*, *C. ovatum*, *C. polyadelphum* and *C. tonkinense*) growing wild in north central Vietnam were obtained by hydrodistillation, analyzed by gas chromatography and screened for antimicrobial and mosquito larvicidal activity. The leaf essential oil of *C. tonkinense*, rich in β-phellandrene (23.1%) and linalool (32.2%), showed excellent antimicrobial activity (MIC of 32 μg/mL against *Enterococcus faecalis* and *Candida albicans*) and larvicidal activity (24 h LC_50_ of 17.4 μg/mL on *Aedes aegypti* and 14.1 μg/mL against *Culex quinquefasciatus*). *Cinnamomum polyadelphum* leaf essential oil also showed notable antimicrobial activity against Gram-positive bacteria and mosquito larvicidal activity, attributable to relatively high concentrations of neral (11.7%) and geranial (16.6%). Thus, members of the genus *Cinnamomum* from Vietnam have shown promise as antimicrobial agents and as potential vector control agents for mosquitoes.

## 1. Introduction

The Lauraceae is a large family of tropical and subtropical trees and shrubs [1]. In this family, the genus *Cinnamomum* is comprised of around 250 species with concentrations in east and southeast Asia [1]. Vietnam is home to 45 species of *Cinnamomum* [2,3], many of which are used in traditional medicine, for essential oils, as well as for timber [4,5].

We are in the midst of a post-antibiotic era. Numerous pathogenic microorganisms have developed resistance to commonly used antibiotic agents [6,7]. For example, *Klebsiella pneumoniae* [8], *Pseudomonas aeruginosa* [9] and *Staphylococcus aureus* [10], three organisms that are major causes of nosocomial infections, have developed extremely drug resistant (XDR) strains. Likewise, antibiotic resistance is increasing in fungi such as *Candida* ssp. and *Aspergillus* spp. [11]. Essential oils have shown promise as complementary or adjuvant therapies for combating antimicrobial resistance [12,13,14,15,16,17,18,19].

Mosquitoes have been and continue to be the deadliest animals on earth. *Aedes aegypti* (L.) (Diptera: Culicidae) and *Ae. albopictus* (Skuse) are vectors for the arboviral diseases dengue, Zika, chikungunya and yellow fever and *Ae. aegypti* is also a vector for the emerging Rift Valley fever virus [20]. *Culex quinquefasciatus* (Say) is a vector of West Nile virus, Saint Louis encephalitis virus and lymphatic filariasis [21]. *Culex quinquefasciatus* may also serve as a vector in emerging viral diseases such as Zika virus [22], Sindbis virus [23] and Usutu virus [24]. Unfortunately, insecticidal resistance of these mosquito species is increasing leading to failure of vector control programs in many locations [25]. Furthermore, populations of *Ae. aegypti* [26], *Ae. albopictus* [27] and *Cx. quinquefasciatus* [28] are showing widespread resistance to commonly used larvicidal agents. It has been suggested that essential oils may serve as alternative and more ecologically benign mosquito larvicidal agents [29,30,31].

Because of the biological activities and traditional uses of members of the *Cinnamomum* genus, we hypothesize that *Cinnamomum* species from Vietnam may also exhibit potentially useful biological activities. As part of our ongoing investigations into the essential oils of Vietnamese *Cinnamomum* [32,33,34], we have obtained, analyzed and carried out antimicrobial and larvicidal screening of *Cinnamomum ovatum* C.K. Allen (syn. *Cinnamomum rigidissimum* H.T. Chang), *Cinnamomum tonkinense* (Lecomte) A. Chev. (syn. *Cinnamomum albiflorum* var. *tonkinense* Lecomte), *Cinnamomum damhaensis* Kosterm., *Cinnamomum longipetiolatum* H.W. Li and *Cinnamomum polyadelphum* (Lour.) Kosterm. (syn. *Laurus polyadelpha* Lour., *Cinnamomum litseafolium* Lecomte, *Cinnamomum litseafolium* var. *denticupulatum* Liou, *Cinnamomum saigonicum* Farw, *Camphorina saigonica* Farw).

## 2. Results

### 2.1. Essential Oil Collection and Analysis

Plant materials were collected from mature *Cinnamomum* trees from different locations in north central Vietnam. The collection details and essential oil yields of the *Cinnamomum* species are summarized in Table 1. The essential oils were analyzed by gas-chromatography–mass spectrometry (GC-MS) and gas chromatography–flame ionization detector (GC–FID). The chemical compositions of the *Cinnamomum* species are presented in Table 2.

### 2.2. Antimicrobial Screening

The *Cinnamomum* essential oils were screened for antimicrobial activity against Gram-positive (*Enterococcus faecalis*, *Staphylococcus aureus*, *Bacillus cereus*) and Gram-negative (*Escherichia coli*, *Pseudomonas aeruginosa*, *Salmonella enterica*) bacteria and a yeast (*Candida albicans*). Minimum inhibitory concentrations and IC_50_ values were determined using the microbroth dilution assay (Table 3).

### 2.3. Larvicidal Screening

The *Cinnamomum* essential oils were screened for mosquito larvicidal activity against *Aedes aegypti*, *Aedes albopictus* and *Culex quinquefasciatus*. The 24 h and 48 h LC_50_ and LC_90_ values are summarized in Table 4 and Table 5.

## 3. Discussion

### 3.1. Cinnamomum ovatum

The leaf and stem bark essential oils of *C. ovatum* demonstrated broad antimicrobial activity against the organisms tested with MIC values ranging from 16 to 128 μg/mL (Table 3). The major components of the leaf and stem essential oils were eugenol (70.5% and 71.2%, respectively), eugenyl acetate (9.5% and 9.3%, respectively) and linalool (5.9% and 8.3%, respectively) (Table 2). The high concentration of eugenol in these two essential oils is likely responsible for the observed antimicrobial effects. Eugenol has shown broad spectrum antibacterial [35,36] and antifungal [37,38,39] activities. Likewise, the mosquito larvicidal activity of *C. ovatum* leaf essential oil is likely due to eugenol; that compound has shown larvicidal activity against *Ae. aegypti* [40], *Ae. albopictus* [41] and *Cx. quinquefasciatus* [42]. *Cinnamomum cambodianum* leaf [34] and stem bark [33] essential oils from Vietnam have also shown high concentrations of linalool (27.0% and 33.1%, respectively).

### 3.2. Cinnamomum tonkinense

*Cinnamomum tonkinense* leaf essential oil showed excellent antimicrobial activity against *E. faecalis* and *C. albicans* with MIC of 32 μg/mL and good activity against *B. cereus* and *S. aureus* (Table 3). The essential oil is rich in monoterpenes, α-pinene (4.0%), sabinene (3.4%), α-phellandrene (4.8%), β-phellandrene (23.1%), 1,8-cineole (9.8%), linalool (32.2%) (Table 2). Both α-pinene and linalool have shown antibacterial activity against *E. faecalis* [35] and *S. aureus* [43]; α-pinene and 1,8-cineole have shown antifungal activity against *C. albicans* [43]. Sabinene, on the other hand, has shown little [44] or no [45] antimicrobial activity. Likewise, α-phellandrene has shown no activity against *C. albicans* [46]. The leaf essential oils of *C. cordatum* and *C. scortechini* from Pahang, Malaysia, both rich in β-phellandrene (9.0% and 17.3%, respectively) and linalool (17.3% and 16.4%, respectively), have shown antifungal activities against several fungal strains [47].

The leaf essential oil of *C. tonkinense* is one of the most larvicidal in this study (Table 4 and Table 5). The major components in the essential oil likely account for the observed larvicidal activity. α-Pinene, has been shown to be larvicidal against *Ae. aegypti*, *Ae. albopictus* and *Cx. quinquefasciatus* [48]; sabinene and linalool have both demonstrated larvicidal against *Ae. aegypti* and *Cx. quinquefasciatus* [49]; and α-phellandrene has shown activity against *Ae. aegypti* and *Ae. albopictus* [50] as well as *Culex pipiens molestus* [51]. The leaf essential oil of *C. scortechinii*, rich in β-phellandrene (17.3%) and linalool (16.4%), had shown excellent larvicidal activity against *Ae. aegypti* and *Ae. albopictus* (LC_50_ = 21.5 and 16.7 μg/mL, respectively) [52].

### 3.3. Cinnamomum damhaensis

The major components of *C. damhaensis* leaf essential oil were linalool (44.8%) and β-selinene (19.1%) (Table 2). The essential oil also showed pronounced larvicidal activity against *Ae. aegypti* and *Cx. quinquefasciatus* with 48 h LC_50_ values of 17.4 and 18.6 μg/mL, respectively (Table 5), which can be attributed to the high concentration of linalool (see above). Note that *Piper gaudichaudianum* and *Piper humaytanum* leaf essential oils, rich in β-selinene (10.5% and 15.8%, respectively), but devoid of linalool, showed only marginal larvicidal activity against *Ae. aegypti* [53].

### 3.4. Cinnamomum longipetiolatum

The leaf essential oil of *C. longipetiolatum* was dominated by linalool (75.7%, Table 2), which likely accounts for the observed antimicrobial (Table 3) activity; linalool has shown broad antibacterial and antifungal activity [35,54]. Although linalool has shown larvicidal activity against *Ae. aegypti* (LC_50_ = 38.6 μg/mL) and *Cx. quinquefasciatus* (LC_50_ = 42.3 μg/mL) [49], the larvicidal activity of *C. longipetiolatum* leaf oil was less (24 h LC_50_ = 64.2 and 126.8 μg/mL against *Ae. aegypti* and *Cx. quinquefasciatus*, respectively, Table 4).

### 3.5. Cinnamomum polyadelphum

The leaf essential oil of *C. polyadelphum* showed good activity against the Gram-positive organisms tested with MIC values of 32, 64 and 64 μg/mL on *E. faecalis*, *S. aureus* and *B. cereus*, respectively (Table 3). The essential oil also showed notable larvicidal activity against all three mosquito species with 48-h LC_50_ values of 17.3, 20.8 and 11.0 μg/mL against *Ae. aegypti*, *Ae. albopictus* and *Cx. quinquefasciatus*, respectively (Table 5). The major components in *C. polyadelphum* leaf essential oil were camphor (32.2%), neral (11.7%) and geranial (16.6%) (Table 2). The antimicrobial properties of camphor are relatively marginal [55,56]. Citral (mixture of neral and geranial), on the other hand, has shown greater antimicrobial activity on Gram-positive bacteria [57,58,59] and fungi [60,61]. Likewise, citral has exhibited mosquito larvicidal activity against *Ae. albopictus* [62] but camphor is inactive against larvae of *Ae. aegypti*, *Ae. albopictus* [52,62] or *Cx. pipiens* [63].

## 4. Materials and Methods

### 4.1. Plant Material

Leaves or stem bark of the *Cinnamomum* species were collected from locations in north central Vietnam (see Table 1). Plants were identified by Do N. Dai and voucher specimens (Table 1) have been deposited in the plant specimen room, Faculty Agriculture, Forestry and Fishery, Nghe An, College of Economics. The fresh plant materials (2.0 kg each) were shredded and hydrodistilled using a Clevenger apparatus for 4 h to give the essential oils. The essential oil yields are summarized in Table 1.

### 4.2. Gas Chromatographic Analysis

Gas chromatography (GC) analysis was performed on an Agilent Technologies (Santa Clara, CA, USA) HP 7890A Plus Gas chromatograph equipped with a flame ionization detector (FID) and fitted with HP-5ms column (30 m × 0.25 mm, film thickness 0.25 μm, Agilent Technologies). The analytical conditions were—carrier gas H_2_ (1 mL/min), injector temperature (PTV) 250 °C, detector temperature 260 °C, column temperature programmed from 60 °C (2 min hold) to 220 °C (10 min hold) at 4 °C/min. Samples were injected by splitting and the split ratio was 10:1. The volume injected was 1.0 μL. Inlet pressure was 6.1 kPa.

An Agilent Technologies (Santa Clara, California, USA) HP 7890A Plus Chromatograph fitted with a fused silica capillary HP-5ms (30 m × 0.25 mm, film thickness 0.25 μm) and interfaced with a mass spectrometer (HP 5973 MSD) was used for the GC-MS analysis, under the same conditions as those used for GC-FID analysis. The conditions were the same as described above with He (1 mL/min) as carrier gas. The MS conditions were as follows—ionization voltage 70 eV; emission current 40 mA; acquisitions scan mass range of 35–350 amu at a sampling rate of 1.0 scan/s.

The identification of constituents was performed on the basis of retention indices (RI) determined with reference to a homologous series of *n*-alkanes, under identical experimental conditions, co-injection with standards (Sigma-Aldrich, St. Louis, MO, USA) or known essential oil constituents, MS library search (NIST 08 and Wiley 9^th^ Version) and by comparing with MS literature data [64]. The relative amounts of individual components were calculated based on the GC peak area (FID response) without using correction factors.

### 4.3. Antimicrobial Screening

The antimicrobial activity of the essential oils was evaluated using three strains of Gram-positive test bacteria, *Enterococcus faecalis* (ATCC299212), *Staphylococcus aureus* (ATCC25923), *Bacillus cereus* (ATCC14579), three strains of Gram-negative test bacteria, *Escherichia coli* (ATCC 25922), *Pseudomonas aeruginosa* (ATCC27853), *Salmonella enterica* (ATCC13076) and one strain of yeast, *Candida albicans* (ATCC 10231).

Minimum inhibitory concentration (MIC) and median inhibitory concentration (IC_50_) values were measured by the microdilution broth susceptibility assay [65]. Stock solutions of the oil were prepared in dimethylsulfoxide. Dilution series were prepared from 16,384 to 2 μg/mL (2^14^, 2^13^, 2^12^, 2^11^, 2^10^, 2^9^, 2^7^, 2^5^, 2^3^ and 2^1^ µg/mL) in sterile distilled water in micro-test tubes from where they were transferred to 96-well microtiter plates. Bacteria grown in double-strength Mueller-Hinton broth or double-strength tryptic soy broth and fungi grown in double-strength Sabouraud dextrose broth were standardized to 5 × 10^5^ and 1 × 10^3^ CFU/mL, respectively. The last row, containing only the serial dilutions of sample without microorganisms, was used as a positive (no growth) control. Sterile distilled water and medium served as a negative (no antimicrobial agent) control. Streptomycin was used as the antibacterial standard, nystatin and cycloheximide were used as antifungal standards. After incubation at 37 °C for 24 h, the MIC values were determined to be the well with the lowest concentration of agents completely inhibiting the growth of microorganisms. The IC_50_ values were determined by the percentage of microorganisms that inhibited growth based on the turbidity measurement data of EPOCH2C spectrophotometer (BioTeK Instruments, Inc Highland Park Winooski, VT, USA) and Rawdata computer software (Brussels, Belgium) according to the following equations:

(1) $${\%}_{inhibition}=\frac{OD_{control\left(-\right)}-OD_{test\;agent}}{OD_{control\;\left(-\right)}-OD_{control\left(+\right)}}$$

(2) $$IC_{50}=High_{conc}-\frac{\left(High_{inh\%}-50\%\right)\times \left(High_{conc}-Low_{conc}\right)}{\left(High_{inh\%}-Low_{inh\%}\right)}$$

where OD is the optical density, control(–) are the cells with medium but without antimicrobial agent, test agent corresponds to a known concentration of antimicrobial agent, control(+) is the culture medium without cells, High_conc_/Low_conc_ is the concentration of test agent at high concentration/low concentration and High_inh%_/Low_inh%_ is the % inhibition at high concentration/% inhibition at low concentration). Each of the antimicrobial screens were carried out in triplicate.

### 4.4. Larvicidal Screening

Eggs of *Aedes aegypti* were purchased from Institute of Biotechnology, Vietnam Academy of Science and Technology and maintained at the Laboratory of Department of Pharmacy of Duy Tan University, Da Nang, Vietnam. Adults of *Culex quinquefasciatus* and *Aedes albopictus* collected in Hoa Khanh Nam ward, Lien Chieu district, Da Nang city (16°03′14.9″N, 108°09′31.2″E) and were identified by National institute of Malariology, Parasitology and Entomology, Ho Chi Minh City. Adult mosquitoes were maintained in entomological cages (40 × 40 × 40 cm) and fed a 10% sucrose solution and were allowed to blood feed on 1-week-old chicks and mice, respectively. Egg hatchings were induced with tap water. Larvae were reared in plastic trays (24 × 35 × 5 cm). The larvae were fed on Koi fish food. All developmental stages were maintained at 25 ± 2 °C, 65–75% relative humidity and a 12:12 h light:dark cycle at the Laboratory of the Faculty of Environmental and Chemical Engineering of Duy Tan University, Da Nang, Vietnam.

Larvicidal activities of the *Cinnamomum* essential oils were evaluated according to the protocol Liu and co-workers [66] with slight modifications. For the assay, 150 mL of water that contained 20 larvae (fourth instar) was placed in 250-mL beakers and aliquots of the *Cinnamomum* essential oils dissolved in EtOH (1% stock solution) were then added. With each experiment, a set of controls using EtOH only (negative control) and permethrin (positive control) were also run for comparison. Mortality was recorded after 24 h and again after 48 h of exposure during which no nutritional supplement was added. The experiments were carried out at 25 ± 2 °C. Each test was conducted with four replicates with five concentrations (100, 50, 25, 12.5 and 6 μg/mL). The data obtained were subjected to log-probit analysis [67] to obtain LC_50_ values, LC_90_ values and 95% confidence limits using Minitab^®^ 19 (Minitab, LLC, State College, PA, USA).

## 5. Conclusions

The essential oils of five species of *Cinnamomum* were collected from north central Vietnam and screened for antimicrobial and mosquito larvicidal activities. According to Duarte and co-workers [68], essential oils with MIC values between 50 and 500 μg/mL can be considered to have strong antimicrobial activity. Similarly, Dias and Moraes have concluded that essential oils with LC_50_ < 100 μg/mL are considered to be active [69]. Therefore, all of the *Cinnamomum* essential oils in this study can be considered to be active and show promise as antimicrobial agents and as alternative insecticidal agents against mosquito larvae.

## Figures and Tables

**Table 1:** Collection details for *Cinnamomum* species from north central Vietnam.

Cinnamomum Species	Vietnamese Name	Voucher Numbers	Part	Yield, % v/w	Collection Month/Year	Collection Location
*Cinnamomum ovatum*	Re trứng	DND-762	Leaf Stems	0.60 0.21	April/2019	Chau Hoan Commune, Pù Huống Nature Reserve 19°28′12″N, 104°56′45″E, elev. 374 m
*Cinnamomum tonkinense*	Re xanh, Re bắc, Quế bắc	DND-768	Leaf	0.33	April/2019	Chau Hoan Commune, Pù Huống Nature Reserve 19°28′12″N, 104°56′45″E, elev. 374 m
*Cinnamomum damhaensis*	Re đầm hà	DND-786	Leaf	0.30	July/2019	Huong Phu Commune, Nam Đông District, Bach Ma National Park 16°12′47″N, 107°43′33″E, elev. 101 m
*Cinnamomum longipetiolatum*	Re cuống dài	DND-800	Leaf	1.35	August/2019	Nam Nhong Commune, Que Phong District, Pù Hoạt Nature Reserve 19°30′24″N, 104°42′52″E, elev. 667 m
*Cinnamomum polyadelphum*	Quế bời lời, Miếng sành, Tà Dúi, Ô dược, Đam dao, Hậu phát	DND-813	Leaf	1.20	August/2019	Nam Nhong Commune, Que Phong District, Pù Hoạt Nature Reserve 19°30′24″N, 104°42′52″E, elev. 667 m

**Table 2:** Chemical compositions (%) of *Cinnamomum* essential oils from north central Vietnam.

N^o^	Compounds	RI^a^	RI^b^	*C. ov.* ^c^	*C. ov.* ^c^	*C. to.* ^d^	*C. da.* ^e^	*C. lo.* ^f^	*C. po.* ^g^
				Leaf	Stem	Leaf	Leaf	Leaf	Leaf
1	α-Thujene	930	924	-	-	0.4	-	-	0.3
2	α-Pinene	939	932	2.1	1.6	4	0.3	2.9	4.3
3	α-Fenchene	953	945	-	-	-	0.1	-	0.2
4	Camphene	955	946	0.7	0.6	0.4	0.2	0.3	1.9
5	Sabinene	978	969	-	-	3.4	0.7	0.5	0.4
6	β-Pinene	984	974	0.9	0.6	2.1	0.2	1.7	2.4
7	Myrcene	992	988	0.2	0.1	3.1	0.1	0.2	2.1
8	Dehydroxy-*trans*-linalool oxide	995	991	-	-	-	-	0.7	-
9	Dehydroxy-*cis*-linalool oxide	1008	1006	-	-	-	-	0.6	-
10	α-Phellandrene	1010	1002	1.3	0.3	4.8	-	-	0.2
11	α-Terpinene	1022	1014	-	-	0.4	-	0.2	
12	*p*-Cymene	1030	1020	0.7	0.4	0.5	0.7	0.2	0.7
13	Limonene	1035	1024	0.9	0.8	3.4	0.2	0.3	5.4
14	β-Phellandrene	1036	1025	-	-	23.1	-	-	-
15	1,8-Cineole	1038	1026	0.2	0.6	9.8	1	2.5	0.8
16	(*E*)-β-Ocimene	1049	1044	0.3	0.2	0.3	-	0.8	-
17	γ-Terpinene	1063	1054	-	-	0.5	-	0.3	-
18	Terpinolene	1094	1086	0.2	0.1	0.2	-	-	-
19	Rosefuran	1098	1091	-	-	-	-	-	0.1
20	Perillene	1104	1102	-	-	-	-	-	0.2
21	Linalool	1105	1095	5.9	8.3	32.2	44.8	75.7	3.2
22	Hotrienol	1107	1104	-	-	-	-	3.2	-
23	Isocitral	1147	1140	-	-	-	-	-	0.2
24	Camphor	1156	1141	-	-	-	-	-	32.2
25	Nerol oxide	1158	1154	-	-	-	-	0.2	-
26	Isoneral	1166	1162	-	-	-	-	-	0.6
27	*cis*-Linalool oxide (pyranoid)	1174	1170	-	-	-	-	3.2	-
28	*trans*-Linalool oxide (pyranoid)	1177	1173	-	-	-	-	2.7	-
29	Borneol	1178	1165	-	-	0.2	-	-	1.6
30	Isogeranial	1184	1180	-	-	-	-	-	0.9
31	Terpinen-4-ol	1187	1174	-	-	0.7	0.4	0.5	0.4
32	α-Terpineol	1200	1186	-	-	1.7	0.2	0.6	0.6
33	Decanal	1208	1201	-	-	0.2	-	-	0.2
34	Citronellol	1228	1223	-	-	-	-	-	0.4
35	Nerol	1231	1227	-	-	-	-	-	0.8
36	Cuminal	1238	1238	-	-	-	0.1	-	-
37	Neral	1245	1235	-	-	-	-	-	11.7
38	Geraniol	1255	1249	-	-	-	-	0.2	1.9
39	Geranial	1274	1264	-	-	-	-	-	16.6
40	(*E*)-Cinnamaldehyde	1278	1267	-	-	-	-	-	0.2
41	Bornyl acetate	1294	1287	0.5	0.5	0.1	-	-	0.1
42	Safrole	1299	1285	-	0.2	-	-	-	-
43	δ-Elemene	1348	1335	-	-	0.2	-	-	-
44	Eugenol	1367	1356	70.5	71.2	0.2	-	-	0.4
45	α-Ylangene	1385	1373	0.4	0.3	-	-	-	-
46	α-Copaene	1389	1374	-	-	0.4	-	-	0.1
47	β-Elemene	1403	1389	-	-	0.3	0.7	-	-
48	Methyl eugenol	1409	1403	-	0.3	-	0.1	-	0.3
49	β-Caryophyllene	1437	1417	1.9	1	1.8	0.2	-	1.3
50	*trans*-α-Bergamotene	1445	1432	0.2	0.2	0.1	-	-	-
51	*allo*-Aromadendrene	1457	1458	0.1	-	-	0.3	-	-
52	α-Humulene	1471	1452	0.3	-	0.6	-	-	0.2
53	α-Amorphene	1483	1483	-	-	-	0.3	-	-
54	β-Selinene	1489	1489	-	-	-	19.1	-	-
55	*trans*-β-Bergamotene	1496	1480	-	0.1	-	-	-	-
56	α-Selinene	1498	1498	-	-	-	0.5	-	-
57	Germacrene D	1498	1484	0.3	0.1	2.7	-	-	-
58	Bicyclogermacrene	1513	1500	0.7	0.6	1	-	0.2	0.2
59	β-Bisabolene	1517	1505	-	0.2	-	-	-	-
60	Eugenyl acetate	1533	1521	9.5	9.3	0.1	-	-	-
61	δ-Cadinene	1537	1522	0.2	0.3	0.2	-	-	0.1
62	(*E*)-α-Bisabolene	1551	1544	-	0.3	-	-	-	-
63	(*E*)-Nerolidol	1570	1561	-	-	0.3	0.9	-	-
64	Germacrene B	1577	1559	-	-	0.2	-	-	-
65	Spathulenol	1598	1577	-	0.6	-	0.4	1.1	0.8
66	Caryophyllene oxide	1605	1582	-	0.5	0.2	1.1	0.5	0.9
67	Intermedeol isomer	1616	-	-	-	-	5.8	-	-
68	Selin-11-en-4-one	1626	1626	-	-	-	1.5	-	-
69	Selina-3,11-dien-6α-ol	1644	1642	-	-	-	0.6	-	-
70	α-Cadinol	1652	1652	-	-	-	0.5	-	-
71	Selin-11-en-4α-ol	1660	1658	-	-	-	7.3	-	-
72	Germacra-4(15),5,10(14)-trien-1α-ol	1685	1685	-	-	-	1	-	-
73	Aromadendrane-4,10-diol	1717	-	-	-	-	0.5	-	-
74	Oplopanone	1735	1739	-	-	-	0.3	-	-
75	α-Cyperone	1747	-	-	-	-	4	-	-
76	Cyclocolorenone	1763	1759	-	-	-	0.1	-	-
	Monoterpene hydrocarbons			7.3	4.7	46.6	2.5	7.4	17.9
	Oxygenated monoterpenoids			6.6	9.4	44.7	46.5	90.1	72.3
	Sesquiterpene hydrocarbons			4.1	3.1	7.5	21.1	0.2	1.9
	Oxygenated sesquiterpenoids			0	1.1	0.5	24	1.6	1.7
	Phenylpropanoids			80	81	0.3	0.1	0	0.9
	Others			0	0	0.2	0	0	0.2
	Total identified			98	99.3	99.8	94.2	99.3	94.9

^a^ RI = Retention Index determined on an HP-5ms column. ^b^ RI from the databases. ^c^ *C. ov.* = *Cinnamomum ovatum*. ^d^ *C. to.* = *Cinnamomum tonkinense*. ^e^ *C. da.* = *Cinnamomum damhaensis.* ^f^ *C. lo.* = *Cinnamomum longipetiolatum.* ^g^ *C. po.* = *Cinnamomum polyadelphum.*

**Table 3:** Antimicrobial activities of *Cinnamomum* essential oils from north central Vietnam.

	Gram-Positive			Gram-Negative			Yeast
Sample	*Enterococcus faecalis*	*Staphylococcus aureus*	*Bacillus cereus*	*Escherichia coli*	*Pseudomonas aeruginosa*	*Salmonella enterica*	*Candida albicans*
	**MIC (μg/mL^a^)**						
*C. ovatum* leaf	64	64	128	64	128	64	64
*C. ovatum* stem	64	64	64	64	16	64	32
*C. tonkinense*	32	128	128	-	-	-	32
*C. damhaensis*	-	-	-	-	-	-	-
*C. longipetiolatum*	64	128	128	256	256	128	256
*C. polyadelphum*	32	64	64	-	-	128	256
Streptomycin	32	128	64	32	128	64	-
Nistatin	-	-	-	-	-	-	8
Cyclohexamide	-	-	-	-	-	-	32
	**IC_50_ (μg/mL^a^)**						
*C. ovatum* leaf	32.33	32.33	65.45	32.56	65.44	33.22	33.22
*C. ovatum* stem	32.44	32.78	33.56	32.56	8.77	31.22	15.67
*C. tonkinense*	5.67	37.78	56.67	-	-	-	15.67
*C. damhaensis*	-	-	-	-	-	-	-
*C. longipetiolatum*	17.88	36.78	56.79	100.34	105.67	56.78	112.45
*C. polyadelphum*	10.67	24.78	30.24	-	-	57.45	123.45

^a^ Micrograms of essential oil per milliliter of test solution.

**Table 4:** Twenty-four-hour mosquito larvicidal activities (μg/mL) of *Cinnamomum* essential oils from north central Vietnam.

Sample	LC_50_ (95% Confidence Limits)	LC_90_ (95% Confidence Limits)	χ^2^	*p*
	*Aedes aegypti*			
*C. ovatum* leaf EO	24.12 (20.92–27.45)	50.61 (45.02–58.65)	48.86	0.000
*C. ovatum* stem EO	52.51 (48.77–57.69)	71.23 (64.50–82.64)	0.4722	0.790
*C. tonkinensis* leaf EO	17.44 (15.53–19.58)	31.40 (27.93–36.64)	0.1354	0.987
*C. damhaensis* leaf EO	21.43 (18.66–24.15)	38.98 (34.75–45.58)	0.5494	0.760
*C. longepetiolatum* leaf EO	64.20 (55.67–73.61)	127.9 (111.0–156.5)	8.805	0.003
*C. polyadelphum* leaf EO	23.41 (21.37–25.78)	36.69 (33.27–41.52)	8.277	0.041
	*Aedes albopictus*			
*C. ovatum* leaf EO	n.t.	n.t.	---	---
*C. ovatum* stem EO	61.45 (55.66–68.20)	103.3 (93.3–117.1)	34.38	0.000
*C. tonkinensis* leaf EO	42.89 (39.73–46.59)	61.65 (56.52–69.09)	2.595	0.273
*C. damhaensis* leaf EO	43.91 (41.25–46.46)	56.16 (52.95–60.79)	0.04480	0.978
*C. longepetiolatum* leaf EO	n.t.	n.t.	---	---
*C. polyadelphum* leaf EO	20.66 (18.02–23.28)	37.21 (33.04–43.97)	2.577	0.276
	*Culex quinquefasciatus*			
*C. ovatum* leaf EO	34.19 (31.18–37.65)	56.01 (50.85–63.12)	10.73	0.013
*C. ovatum* stem EO	28.79 (22.07–34.79)	78.3 (67.72–94.57	8.295	0.016
*C. tonkinensis* leaf EO	14.05 (12.28–15.75)	25.70 (23.06–29.59)	16.31	0.001
*C. damhaensis* leaf EO	46.74 (41.58–52.63)	86.80 (77.37–100.39)	13.53	0.001
*C. longepetiolatum* leaf EO	126.8 (108.3–151.4)	293.9 (248.2–368.9)	21.47	0.000
*C. polyadelphum* leaf EO	18.33 (13.26–22.87)	58.95 (50.62–72.05)	5.639	0.131

**Table 5:** Forty-eight-hour mosquito larvicidal activities (μg/mL) of *Cinnamomum* essential oils from north central Vietnam.

Sample	LC_50_ (95% Confidence Limits)	LC_90_ (95% Confidence Limits)	χ^2^	*p*
	*Aedes aegypti*			
*C. ovatum* leaf EO	13.76 (11.42–15.95)	30.17 (26.76–35.17)	46.16	0.000
*C. ovatum* stem EO	46.74 (43.17–51.21)	67.53 (61.30–77.07)	2.744	0.254
*C. tonkinensis* leaf EO	15.83 (13.76–17.99)	31.17 (27.53–36.80)	2.196	0.533
*C. damhaensis* leaf EO	17.36 (13.67–20.36)	37.53 (32.94–45.22)	5.494	0.064
*C. longepetiolatum* leaf EO	39.50 (29.92–47.24)	95.24 (83.27–114.77)	2.513	0.113
*C. polyadelphum* leaf EO	17.30 (15.44–19.41)	30.80 (27.43–35.89)	3.650	0.302
	*Aedes albopictus*			
*C. ovatum* leaf EO	n.t.	n.t.	---	---
*C. ovatum* stem EO	50.18 (45.07–56.12)	87.98 (78.88–100.81)	35.66	0.000
*C. tonkinensis* leaf EO	42.74 (39.48–46.59)	62.40 (57.00–70.25)	4.098	0.129
*C. damhaensis* leaf EO	39.85 (37.05–42.91)	56.02 (51.91–61.70)	0.06006	0.970
*C. longepetiolatum* leaf EO	n.t.	n.t.	---	---
*C. polyadelphum* leaf EO	20.79 (17.84–23.61)	39.45 (34.97–46.59)	6.980	0.031
	*Culex quinquefasciatus*			
*C. ovatum* leaf EO	30.48 (27.00–34.48)	59.19 (52.54–68.81)	1.181	0.757
*C. ovatum* stem EO	20.54 (11.92–27.10)	72.40 (62.01–89.54)	5.799	0.055
*C. tonkinensis* leaf EO	8.721 (6.874–10.253)	18.81 (16.70–22.01)	26.83	0.000
*C. damhaensis* leaf EO	18.63 (9.90–25.06)	67.93 (58.16–84.15)	6.243	0.001
*C. longepetiolatum* leaf EO	76.88 (52.08–101.93)	314.5 (249.3–447.0)	47.36	0.000
*C. polyadelphum* leaf EO	11.03 (4.50–15.93)	52.40 (44.35–65.76)	10.30	0.016
